# Intervention Based on Psychomotor Rehabilitation in Children with Autism Spectrum Disorder ASD: Effect on Postural Control and Sensory Integration

**DOI:** 10.3390/children10091480

**Published:** 2023-08-30

**Authors:** Imen Ben Hassen, Rihab Abid, Fatma Ben Waer, Liwa Masmoudi, Sonia Sahli, Tarak Driss, Omar Hammouda

**Affiliations:** 1 Research Laboratory, Molecular Bases of Human Pathology, LR19ES13, Faculty of Medicine, University of Sfax, Sfax 3026, Tunisia; imenbenhassen@live.fr (I.B.H.); omar.hammouda@parisnanterre.fr (O.H.); 2 Research Unit, Physical Activity, Sport and Health, UR18JS01, National Observatory of Sport, Tunis 1003, Tunisia; isseps.rihababid@gmail.com; 3 Research Laboratory, Education Motricité Sport et Santé EM2S LR19JS01, High Institute of Sport and Physical Education of Sfax, University of Sfax, Sfax 3000, Tunisia; fatmaelwaer123@gmail.com (F.B.W.); liwa.masmoudi@isseps.usf.tn (L.M.); sonia.sahli.isseps@gmail.com (S.S.); 4 Interdisciplinary Laboratory in Neurosciences, Physiology and Psychology: Physical Activity, Health and Learning (LINP2), UPL, UFR STAPS, Paris Nanterre University, 92001 Nanterre, France

**Keywords:** psychomotricity, children, ASD, postural control, vision

## Abstract

Postural stability and control are essential motor skills for successfully performing various activities of daily living. However, children with autism spectrum disorder (ASD) exhibit significant sensorimotor impairments. The aim of this study was to investigate the efficacy of psychomotricity training on postural control (PC) of children with ASD. We recruited thirty children (age = 8.01 ± 1.2; weight = 31.66 ± 8.1 kg; height = 129.7 ± 10.8 cm) diagnosed with ASD (intellectual quotient > 50) to participate in this study. They were divided into two groups: the experimental group (n = 16) and control group (n = 14). Children in the experimental group were trained with psychomotor activities two times a week for nine weeks. Statistic postural balance was assessed before and after intervention and on different vision conditions. The results showed that the psychomotor training significantly improved PC in standing position under different conditions when compared to the control group, in all parameters (CoP_A_; CoP_LX_; CoP_Ly_) (*p* < 0.01). Our preliminary findings suggest the usefulness of the psychomotor training in children with ASD on static PC.

## 1. Introduction

Autism spectrum disorder (ASD) has been listed as one of the most prevalent developmental disabilities in the world; it begins in childhood and tends to persist into adolescence and adulthood [1]. In 2020, the Centers for Disease Control and Prevention reported that approximately 1 in 54 children is diagnosed with ASD, according to 2016 data based on tracking within 11 communities in the United States [2]. Children with ASD suffer from deficits in social communication and interaction associated with restricted and repetitive patterns of behavior [3]. In addition, 80% of children diagnosed with ASD showed problems with motor coordination and skills [4,5], deficits in gross and fine motor skills [6] clumsiness, reduced ankle movement [7], and abnormal gait pattern (walk on tiptoes) [8]. Furthermore, they often show impaired static and dynamic postural balance [9]. On the other hand, abnormalities in the sensory process were detected in 90% of ASD population that show hypo- or hyper-sensory reaction [10]. Consequently, sensory impairments were included as part of the fundamental definition of autism disorders [11].

Moreover, earlier research provides evidence for the association between sensory processing problems and motor impairments in children with ASD starting from the first years of life [12]. These findings highlight the need to take into account both sensory and motor domains when evaluating and developing rehabilitation programmes for individuals with ASD. The specific mechanisms underlying this association are complex and multifaceted and vary among individuals. ASD‘s sensory–motor impairments may be attributed to the abnormal neural connectivity between sensory processing areas and motor control regions, leading to difficulties in processing sensory information and translating it into coordinated motor responses [13]. In addition, sensory processing issues in children with ASD, such as under/over responsivity to sensory stimuli, can disrupt the integration of sensory information necessary for motor control, which can alter motor abilities [8]. Thus, there is a critical need to promote an effective intervention of physical activity in order to minimize the motor dysfunction in ASD.

Several researches have focused on the measurement of postural control (PC) in ASD children and have reported increased sway during static [9,14] and dynamic [15,16] postural balance. It is evidenced that PC involves cues from visual, vestibular, and proprioceptive channels [17]. During sensory stimulation, postural sway increased in response to changes in sensory stimuli [18] as a result of adjustments within the PC system to maintain an upright posture [19]. Yet, children with ASD are reported to have problems in modulating sensory information and deficiencies in visual processing [20,21]. It is obvious that postural sway increased when visual information was unavailable in ASD children [22]. Stins et al. [23] also reported that children with mild ASD had increased postural instability in the mediolateral direction while their eyes were closed (EC). These authors suggested that individuals with ASD depend more on visual input for the regulation of balance, which makes PC maintenance more difficult with EC [23].

Based on these autism-related complications and given the importance of sensory integration cues for maintaining balance in ASD, physical activity (PA) interventions seems to be one of the most beneficial and regulatory methods to minimize balance deficit [24]. Most previous studies suggest that PA interventions can positively impact motor abilities, coordination, and balance in children with ASD [25]. However, evidence on the efficacy and feasibility of psychomotor rehabilitation for children affected by ASD is lacking [26].

Despite the importance of developing the sensorimotor system in children with ASD, only a few studies are available about the potential beneficial effect of PA on PC in this population. The existing data have reported an improvement in PC in response to different PA interventions, including visual-based biofeedback [27], horse riding [28], swimming [29], and Taekwondo [30]. For example, Cheldavi et al. [24] have found a significant improvement in all postural parameters in trained ASD children in all conditions (firm/foam surface; open/closed eyes) after six weeks of a balance rehabilitation program. Moreover, another previous study found an improvement in standing balance in both the right and left leg with eyes open (EO) and EC in ASD children following 10-week swimming training [31] A recent study from Caldani et al. [24,30,32] explored the effect of a short postural rehabilitation training program in twenty children with ASD and demonstrated beneficial results on postural stability.

Psychomotor interventions have shown positive results as therapeutic approaches which have demonstrated benefits for children with attention deficit hyperactivity disorder [33]. Indeed, psychomotor rehabilitation may be considered as a safe and efficacious therapy for many neurodevelopmental disorders [34,35]. However, limited studies have been performed on the ASD population, especially in children [26,35,36]. In this context, more rigorous research concerning psychomotor rehabilitation is required to understand the benefits, effectiveness, and ideal approaches of such interventions for this specific population.

Given the complexity of the impairments related to ASD, different rehabilitative and training approaches have been proposed to ameliorate PC [24,30,32]. However, there is no consensus on the effect of psychomotor rehabilitation in PC on ASD children.

According to these findings, the aim of the present study was to verify if an intervention program touching on muscle output responses and the psychic side could improve the balance deficit in Tunisian children with ASD. In this context, we hypothesized that such intervention could have beneficial effects on the PC of children with ASD.

## 2. Materials and Methods

### 2.1. Participants

Thirty children (age: 8.06 ± 1.25 years, weight: 31.66 ± 8.1 kg, height: 129.7 ± 10.8 cm) with ASD and enrolled from the local autistic care centre were selected for this study and were randomly allocated to either a control group (ASD Ctrl) (n = 14) or an experimental group (ASD T) (n = 16). The two groups were matched in age, sex, and intellectual quotient (IQ) to ensure good reliability. The mean age and anthropometric characteristics of the participants are shown in Table 1. All participants have a confirmed diagnosis of ASD by a licensed professional (e.g., a physician or psychologist). Each child had to meet the criteria of ASD diagnosis on both the DSM-V (Association and DSM-V, 2013) and childhood autism rating scale (CARS) [37]. This latter scale was used by a psychiatrist to assess the severity of the children’s ASD symptoms [34,35]. All participants in the study had CARS scores between 30 and 37 indicating mild to moderate autism] [37]. Inclusion criteria: (1) diagnosed with mild to moderate level of ASD; (2) had the ability to understand and communicate and to perform motor tasks. The exclusion criteria: chronic medical disorders, visual and/or physical impairments, attention deficit hyperactivity disorder, neurological disorders (i.e., individuals with cerebral palsy, epilepsy, neuromuscular disorders, and tethered spinal cord), and intellectual disability. During the intervention period, all children did not attend any additional exercise training in or out of school. They were asked to maintain their habitual rhythm of being asleep/awake. Parents of ASD children provided written informed consent before starting experimental procedures. The present study was conducted in accordance with the Declaration of Helsinki and was approved by the local Institutional Review Board (CPP SUD N°0134/2018).

### 2.2. Experimental Procedure

The experiment and test sessions took place in a physical activity room (6 m long, 3 m wide) of a center of care of ASD. The experimental protocol consisted of the evaluation of the effects of psychomotricity training on postural balance for children with ASD. Participants realized 2 different vision situations: a bipedal stance with EO and EC.

All measurements were performed in the morning to control the effect of circadian rhythm on postural balance [38]. A familiarization session was performed before the experimental protocol execution to eliminate the fear of new materials used and to ensure high-quality results. Each participant was asked to maintain a standing upright position on a stabilometric platform with their arms resting at their sides for 25 s in front of the picture located 1 m away from him/her. Foot placement was marked on the force plates to facilitate standing for all trials. During the EC tasks, subjects were not able to keep their EC—that was why they were blindfolded. The experimenter remained near them for security reasons without providing additional help. Verbal instructions were simple and standardized in order to minimize any confounding elements of language and comprehension.

**Figure 1:**
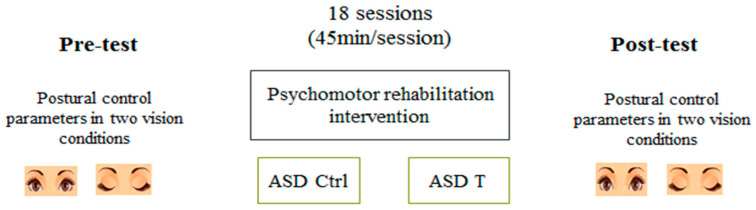
Study design.

The psychomotor training consisted of two training sessions per week for nine weeks. Each training session lasted approximately 45 min and was composed of four parts: 5 min warming up, then 15 min of trampoline activity followed by 20 min of psychomotricity exercises in circuit form, and finally 5 min of cool-down and stretching (Figure 1). Psychomotricity rehabilitation aimed to develop the integration between cognition (psycho) and movement (motor) [39]. It was based on different core areas: body awareness, spatial awareness, directional awareness, temporal awareness, and rhythm [40]. Therefore, our training program involved balancing activities, cognitive stimulation games, fine and gross motor exercise, and jumping and sensory integration activities (Table 2).

The experimental group members took part in 18 training sessions of the program, whereas the control group members were conducting their regular center schedule.

Furthermore, all participants were invited to the physical activity room during three sessions, namely a familiarization session and two test sessions (a pre- and post-testing sessions) in the morning to control the effect of circadian rhythm on postural balance [38].

The familiarization session was performed before the experimental protocol execution to eliminate the fear of new materials used and ensures high quality of results. During this session, participants were familiarized with the experimental protocol, including the evaluation of their postural balance. The pre- and post-testing sessions were performed two days before and after the intervention program, in which all participants’ postural balance was assessed in a bipedal stance with both EO and EC conditions.

### 2.3. Postural Measurements

Postural balance was evaluated using a stabilometric platform (PostureWin ©, Techno Concept^®^, Cereste, France; 40 Hz frequency, 12-bit A/D conversion), composed of a steel plate supported by three tri-axial transducers, to record the participants’ center of pressure (CoP) excursions (Normes 85; Association Française de Posturologie, 1985).

Participants were asked to stand as still as possible on the stabilometric platform in a bipedal position with their arms comfortably placed along the body. Their bare feet were separated by an angle of 30° and their heels were placed 5 cm apart. Postural balance measurements were performed in two different vision conditions (EO/EC) (Figure 1).

During the EO condition, participants were asked to look straight ahead at a red cross placed onto the wall, 2 m away at eye level, whereas in the EC condition, they wore a blindfold and were instructed to keep their gaze horizontal in a straight-ahead direction (Figure 1). Each experimental condition was tested with three trials where each one lasted 30 s with a 30 s resting period in between. The best measurement was considered for analysis. The experimenter remained near them for security reasons without providing additional help. Verbal instructions were simple and standardized in order to minimize any confounding elements of language and comprehension.

The CoP excursions were evaluated from the ground reaction forces and they represent the muscular torque that controls the oscillations of the body. During a bipodal position, the human body oscillates and this leads to variations in companion muscle torque, mainly depending on the CoP excursions [41]. To evaluate postural balance, the surface area and the lengths of the CoP parameters were selected. The surface of the CoP corresponds to an ellipse with 90% of CoP excursions and, thus, is considered as an index of the overall postural performance [41,42]. The CoP length corresponds to the sum of CoP displacement in the mediolateral (CoP_LX_) and anteroposterior (CoP_LY_) directions.

### 2.4. Statistical Analysis

All statistical analyses were processed using STATISTICA 10 Software (StatSoft, Maisons-Alfort, France). The normality of the data was verified by the Shapiro–Wilk test. Demographic data including age, weight, and height were expressed as means ± standard deviation (SD). An independent t-test was used to compare age, weight, height, IQ, and CARS score between both groups. Three-way ANOVA (group × surface × vision) with repeated measures was used to determine the effect of group (ASD Ctrl vs. ASD T), vision (EO vs. EC), and training (before vs. after) factors on the dependent variables (CoP_A_; CoP_LX_; CoP_LY_)_._ A Bonferroni post hoc analysis was used to determine the parameters to which significant differences could be attributed. Correlational analyses were conducted on the CARS scores and postural sway parameters separately for each group and for each condition (before/after; EC/EO) The significance for all analyses was accepted at the level of *p* < 0.05.

## 3. Results

Statistical analyses showed no significant differences between both groups (ASD T and ASD Ctrl) in demographic and baseline characteristics, including age, weight, height, and CARS score, as shown in Table 1.

In addition, three-way ANOVA (group × training × vision) indicated no significant effects between groups in CoP_Ly_ (*p* > 0.05). There was a significant effect of visual conditions in all postural parameters: CoP_A_ (F_(1,28)_ = 159.16, *p* < 0.01), CoP_Lx_ (F_(1,28)_ = 33.97, *p* < 0.01), and CoP_Ly_ (F_(1,28)_ = 20.85, *p* < 0.01).

Moreover, three-way ANOVA indicated a significant effect of training intervention on all postural parameters: CoP_A_ F_(1,28)_ = 104.96, *p* < 0.01), CoP_Lx_ (F_(1,28)_ = 27.72, *p* < 0.01), and CoP_Ly_ F_(1,28)_ = 24.42, *p* < 0.01). A significant interaction was observed between training and group factors (*p* < 0.01). However, no significant interaction between vision, training and group was found (Table 3).

**Figure 2:**
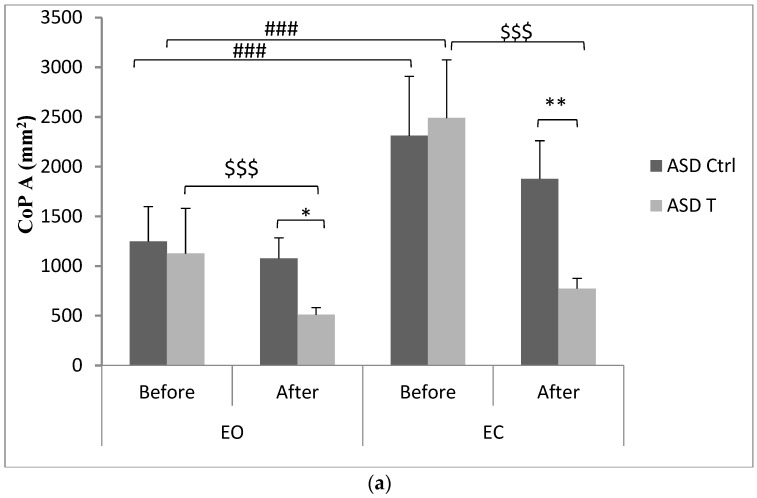

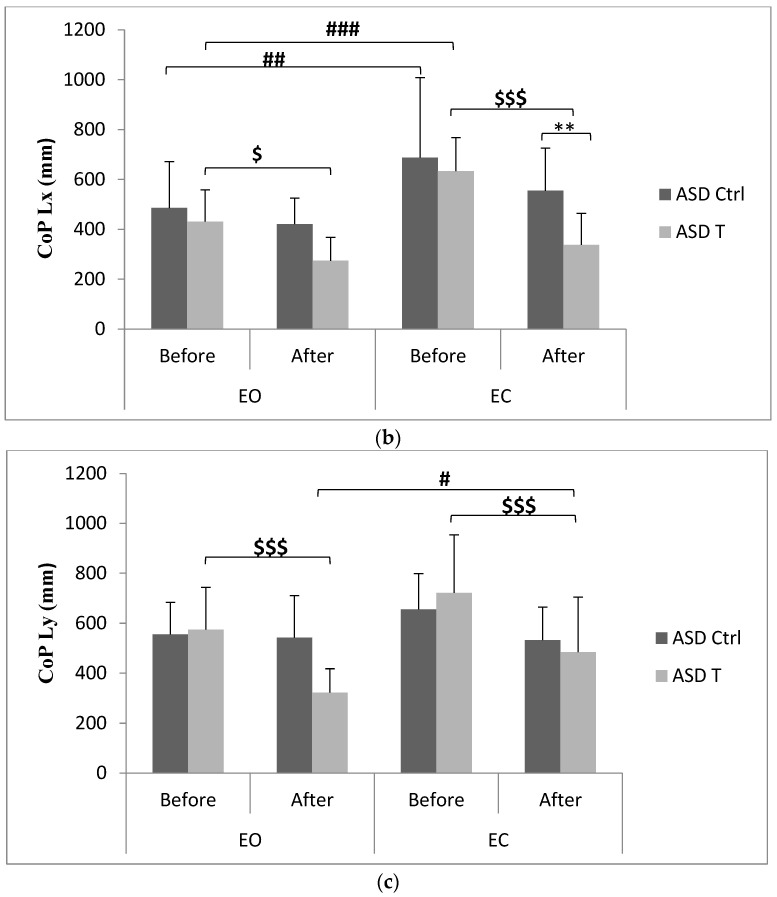
Mean values and standard deviations of CoP A (mm^2^) (**a**) CoP _Lx_ (mm); (**b**) CoP _Ly_ (mm); (**c**) for ASD control (ASD Ctrl) and ASD training (ASD T) groups before and after training in the eyes open (EO) and eyes closed (EC). *, **: significantly different from ASD Ctrl at *p* < 0.05 and *p* < 0.01, respectively; #, ##, ###: significantly different from EO at *p* < 0.05, *p* < 0.01, and *p* < 0.001, respectively; $, $$$: significantly different from pre-training at *p* < 0.05, *p* < 0.001 respectively.

Bonferroni results showed that the CoP values (CoP_A_, CoP_Lx_, CoP_Ly_) significantly decreased in post-training session compared to pre-training session only for the training group. Conversely, the control group showed no significant changes (Figure 2).

Concerning the vision effect, the Bonferroni test showed that in the EC condition, CoP values were significantly increased compared to EO in both groups. 

For the correlation results, the Spearman analysis revealed that in the ASD T group there was a negative significant correlation (*p* < 0.01) between CARS score and CoP _A_ in the EC condition after training (r = −0.712; *p* = 0.002).

## 4. Discussion

The present study aimed to investigate the effect of 9-week psychomotricity training in children with ASD on PC. The present findings showed significant improvements in all PC parameters measured after the psychomotor rehabilitation.

Despite numerous studies showing improvements in motor skills in ASD children [43,44,45,46], there is an overall lack of interventions aiming to evaluate postural stability after PA interventions [24,27,30]. Previous research showed that postural stability in ASD can be improved through various means, such video games, hippotherapy, and balance training [24,27,47].

Our results are consistent with previous research involving young children with ASD suggesting that swimming intervention reported an improvement in the duration of standing balance on right and left foot under divers sensory conditions [31]. Furthermore, after 20 weeks of horse riding training, ASD children showed improved motor proficiency and sensory integrative functions that persist for at least 24 weeks [48]. However, limited studies have examined the effect of psychomotor rehabilitation for ASD, especially in children [49,50].

Indeed, both training in karate techniques and aquatic exercise programs have a positive influence on static or dynamic balance among ASD children aged between 8 and 14 years [51].

Unfortunately, in previous studies, assessment was used only with qualitative measurement [51]. On the other hand, researchers used a quantitative measure of balance (a force plate) and found a lower sway and more stability for two postural parameters (CoP_Lx_; CoP_Ly_) under two visual conditions after a balance program intervention in ASD children [24]. In addition, our findings are in line with Kim et al [30] showing enhancements in sway velocity in the single leg stance with EC condition following taekwondo training. However, most previous intervention trials used various standardized motor assessments to evaluate score or duration of standing balance (e.g., using the Bruininks–Oseretsky Test of Motor Proficiency, the Pediatric Balance Scale, and a stopwatch). Furthermore, evaluating PC with stabilometric platform could be more objective than other subjective measurements as it helps to obtain quantitative information regarding distribution and oscillations in sway. At the same time, due to the lack of a typical development group, our results should be interpreted with caution.

Studies suggest that appropriate balance is a result of full interaction between neural and biomechanical mechanisms [51,52]. In our study, the improvement in the PC of individuals with ASD could be due to an improvement in body awareness, as previously reported in children with ASD after a psychomotor rehabilitation program [53]. Also, the present findings could be attributable to an improvement in neuromuscular coordination after the intervention, as previously shown after traditional dance training [54]. Martial arts studies showed that balance progress could be related to the following components: cooperation of postural muscular responses, better efficiency in vision, vestibular and somatosensory systems, adaptive systems, improved muscular strength and range of motion, and better physical structure [51,52]. All these segments seem to be related to the achieved result, considering that psychomotor rehabilitation involves non-competitive games and sport-related movements with a focus on individuals’ body, spatial, and temporal sensory-processing competence [55]. Despite the relationship between improved postural balance and other physical performance enhancements in ASD children [56], the measurements of the present study do not assess the target skills and performances promoted in the rehabilitation training.

Even after training, our results showed that the use of sensory information (EC) leads to a significant increase in postural sway. In this context, the greater postural sway in ASD has been detected in various studies in different challenging stances, such as EC conditions [57], one-footed standing [31,58], and standing under different conditions [59,60]. The present findings showed an increased sway in the EC condition for both groups. It is plausible that individuals with ASD display “hyper-reactivity” to sensory disturbances [61]. Moreover, Romberg index EC/EO ratios were <1 in accordance with previous studies [57,60]. This could reflect the importance of visual processing during PC in this population. Indeed, standing impairment in EC justifies the deficiency in PC mechanisms, which altered the integration of visual, proprioceptive, and vestibular systems [62,63]. Also, in this context, a psychomotricity training program relies on different inputs (visual, somatosensory) and leads to more sensory integration and environmental information which can affect the brain activity in children with ASD [64]. According to a recent study, trampolining was therapists’ most frequently recommended sensory-based intervention for ASD children [65]. According to Giagazoglou et al., an intervention of 12-weeks of a trampoline training program resulted in significant improvements in three different balance ability tasks (double-leg stance with EO and EC, and one-leg stance with EO) for intellectual disability (ID) children [66]. Also, a trampoline program for 20 weeks improved postural balance in six ASD children [67]. In general, ASD and ID are two sides of the same coin as neurodevelopmental disorders; however, studies investigating the effect of psychomotor intervention in autism are very limited [49].

In addition, our results revealed that improvements in CoP_A_ and CoP_Lx_ were higher in the EC with respect to EO conditions after training. The findings of the present study can also be compared with similar studies carried out in ASD children. For example, a short postural rehabilitation training program that improved the surface of CoP was higher in EC with respect to EO conditions [32]. These findings have pointed that PC corresponds to a complex neurological function and depends on somatosensory inputs that are transmitted by the visual, proprioceptive, and vestibular systems [68]. From this point of view, using cognitive techniques to give children the chance to increase brain activity and strengthen whole muscle and appropriate sensory integration may present the mechanism that leads to motor skills improvements in ASD subjects [69].

The present findings confirm the results of a recent study showing improvements in static balance tasks by children with ID who participate in a 16-week psychomotor education program [70]. In addition, another study has found an improvement in PC and coordination in the motor competence for young children (aged 4–7 years) with developmental delay including ASD after a psychomotor program [49]. Despite the short duration of the intervention, this study was the first to explore psychomotricity training in young Tunisian children with ASD using an objective measurement of PC in different vision conditions.

We recognize a major limitation of this study. The number of participants is still relatively small. Nevertheless, the findings provide an impetus for conducting future randomized trials with larger sample sizes while controlling for sex differences. Finally, the training program lasted only 9 weeks, suggesting that longer training periods may be necessary to achieve more substantial improvements.

## 5. Conclusions

Favorable results indicate that psychomotor rehabilitation is a relatively effective intervention which could be used as a mean for improving postural sway in different sensory conditions for children with ASD. This knowledge is essential for educators to help children with ASD to develop abilities and skills.

Psychomotor rehabilitation for ASD children can help them to improve their motor skills, including PC in different conditions. These improvements may have a positive impact on daily activities and allow them to perform these activities with greater ease and confidence. In addition, by enhancing motor skills, ASD children may increase their self-confidence, social participation, and emotional well-being. In this context, these findings can be applied in clinical settings to benefit ASD children and help practitioners and scientists in making decisions and devising effective strategies to improve disabilities. They could include developing adaptive psychomotor rehabilitation programs, incorporating PC exercises into therapy sessions, training techniques, and customizing interventions to meet the needs of each patient.

## Figures and Tables

**Table 1:** General characteristics of both groups.

Characteristics	Control Group (n = 14)	Experimental Group (n = 16)	t-Test	*p* Value
Age (years)	8 ± 1.35	8.1 ± 1.204	t = 0.267	*p* = 0.79
Weight (kg)	29.92 ± 10	33.18 ± 9.71	t = 0.90	*p* = 0.37
Height (cm)	127.5 ± 12.2	131.6 ± 9.47	t = 1.04	*p* = 0.30
Intellectual quotient (IQ)	58.64 ± 8.20	61.37 ± 7.57	t = 0.94	*p* = 0.35
CARS score	34.9 ± 1.7	35.12 ± 1.8	t = 0.24	*p* = 0.8

**Table 2:** Psychomotor activities rehabilitation.

Weeks	Performed Activities	Performance Component
1–2 wk	Transferred many bottles of different colors from side A to side B while following a course of obstacles by moving up and down when required.	Body awareness Space awareness Coordination
3–4 wk	Different circuits activities involve jumping in four directions and at different heights. Jumping on one or two legs.	Directional awareness Coordinated movement of all segments of the body
5–6 wk	Walking forward and backward on a balance beam with a tennis ball put on a racket without losing the ball Sliding with the back on a short balance beam with/without using hands or feet.	Dynamic balance Eye-hand coordination
7–9 wk	Going down and up the stairs in a predetermined time. Catching balls from the floor and classifying them according to color. Circuits with different surfaces (fluid and solid). Kicking a ball with the leg into a goal.	Speed Eye-foot coordination Body awareness

**Table 3:** Summary of the statistical results related to postural variables.

	Main Effect									Double Interaction									Triple Interaction		
	Group			Vision			Training			Vision × Group			Training × Group			Vision × Training			Vision × Training × Group		
	F_(1,28)_	*p*	ƞ2p	F_(1,28)_	*p*	ƞ2p	F_(1,28)_	*p*	ƞ2p	F_(1,28)_	*p*	ƞ2p	F_(1,28)_	*p*	ƞ2p	F_(1,28)_	*p*	ƞ2p	F_(1,28)_	*p*	ƞ2p
CoP_A_ (mm^2^)	17.62	<0.001	0.40	159.16	<0.001	0.86	104.96	<0.001	0.80	0.73	0.40	0.03	104.96	<0.001	0.80	37.74	<0.001	0.59	14.23	<0.001	0.35
CoP_Lx_ (mm)	7.21	0.01	0.22	33.97	<0.001	0.57	27.72	<0.001	0.52	0.46	0.51	0.02	27.72	<0.001	0.52	6.13	0.02	0.19	0.71	0.41	0.03
CoP_Ly_ (mm)	0.94	0.34	0.03	20.85	<0.001	0.44	24.42	<0.001	0.48	6.26	0.02	0.19	24.42	<0.001	0.48	1.02	0.32	0.04	1.76	0.20	0.06

CoP_A_: center of pressure (CoP) area, CoP_Lx_: displacement of CoP in the mediolateral axis, CoP_Ly_: displacement of CoP in the anteroposterior axis.

## Data Availability

Data are available on request due to privacy or ethical restrictions.
